# Risk of cervical squamous cell carcinoma associated with a single nucleotide polymorphism in the *RAD18* gene in the Chinese population and its significance as a predictive biomarker

**DOI:** 10.1097/MD.0000000000044017

**Published:** 2025-08-22

**Authors:** Rui Zhang, Jianping Kong, Yun Li, Hanzhi Wang, Qi Cheng, Caiyun Zhou, Minghua Yu, Yitong Wang, Feng Ye

**Affiliations:** a Department of Gynecology and Obstetrics, The First People’s Hospital of Jiande, Hangzhou City, Zhejiang Province, China; b The Second Affiliated Hospital (Jiande Branch), School of Medicine, Zhejiang University, Hangzhou City, Zhejiang Province, China; c Central Laboratory of Women’s Hospital, School of Medicine, Zhejiang University, Hangzhou City, Zhejiang Province, China; d Department of Pathology, Women’s Hospital, School of Medicine, Zhejiang University, Hangzhou City, Zhejiang Province, China; e Neurobiology Major, University of California, San Diego, CA.

**Keywords:** cervical squamous cell carcinoma, genetic predictive biomarker, *RAD18*, single nucleotide polymorphism

## Abstract

*RAD18* is a crucial mismatch repair gene associated with the post-replication repair, and genetic variations in *RAD18* gene are closely related to tumorigenesis. We selected 6 *RAD18* SNP and performed mismatch amplification PCR on 650 cases of CIN III, 580 cervical squamous cell carcinoma (CSCC), and 1320 healthy controls. The *RAD18* rs250403 GG and G allele (AG + GG) genotype risk in CIN III and CSCC were significantly increased. The results showed a significant correlation between the GG genotype of rs615967 and the risk of CIN III and CSCC. Carriers of the G allele (AG + GG) at *RAD18* rs615967 also had an increased risk. More noteworthy was that the *RAD18* rs250403 (A/G) and rs615967 (A/G) haplotypes associated with high risk of CIN III and CSCC were AG-GG, GG-AA, GG-AG, and GG-GG. Clinical data analysis further showed that the polymorphisms of *RAD18* rs250403 and rs615967 were significantly correlated with prognostic indicators such as family history of tumor, differentiation grade, lymph node metastasis, and vascular involvement. *RAD18* protein expression was significantly decreased in CSCCs with the rs615967-AG and rs615967-GG genotype. In summary, the 2 genetic polymorphisms of the *RAD18* were associated with susceptibility and prognosis in CIN III and CSCC, and specific high-risk haplotypes of these 2 SNPs could serve as genetic predictive biomarkers.

**Key Points**

1. Large sample study (580 CSCC and 650 CIN III), high ratio and large scale controls (1:2; 1320 controls) were used in this study, which increased the reliability and repeatability of the results.
2. These findings indicated, for the first time, that 2 SNPs [rs250403 and rs615967] of *RAD18* gene were associated with susceptibility and prognosis of CSCC.
3. The haplotypes of AG-GG, GG-AA, GG-AG, and GG-GG of *RAD18* rs250403 and rs615967 SNP may act as predictive biomarker of CIN III and CSCC.

## 1. Introduction

Cellular DNA is continuously damaged by a range of endogenous and exogenous sources. If not sensed and repaired efficiently, DNA damage leads to genome instability and eventually cancer. The maintenance of genomic integrity is fundamental for cell survival and controlled cell growth. Indeed, most cancer cells exhibit genome instability, often arising from defects in DNA replication defects and faulty repair events.^[1,2]^

Trans-lesion synthesis (TLS) is a DDT mechanism that permits DNA synthesis using templates containing bulky DNA lesions such as ultraviolet (UV)-induced cyclobutane pyrimidine dimers, benzo[a]pyrene dihydrodiol epoxide (BPDE) adducts, 8-oxodG, and many others.^[3]^ DNA damage tolerance (DDT) pathways are largely coordinated by mono- or polyubiquitination of the replicative clamp proliferating cell nuclear antigen (PCNA).^[4,5]^ Mono-ubiquitination of the homotrimeric DNA polymerase processivity factor PCNA contributes to TLS polymerase recruitment at sites of DNA damage.^[6]^ Although several E3 ubiquitin ligases control this modification, *Rad18* is a central regulator, required for both types of PCNA ubiquitination.^[6–9]^

The *RAD18* gene, located on human chromosomes 3p24-p25, plays a crucial role in post-replication repair in various organisms from yeast to humans. Loss of *Rad18* increases mutation rates in cells and sensitizes them to DNA damage, illustrating the importance of the DDT pathways in genome stability and cell survival.^[10,11]^ However, overexpression of Rad18 is also deleterious, as it disrupts the proper assembly of some DNA repair foci^[12]^ and leads to inappropriate PCNA ubiquitination and TLS polymerase recruitment in the absence of DNA damage.^[13]^ These events could perturb DNA repair or processive DNA replication and increase mutagenesis, consistent with the fact that *Rad18* is upregulated in certain cancers.^[14–16]^ Thus, tight control of Rad18 levels and activity promotes genome maintenance.

The genetic variation of the *Rad18* gene is closely related to tumorigenesis. The *RAD18* Arg302Gln polymorphism is associated with the risk of colorectal cancer (CRC).^[17]^ The frequency of RAD18 Gln302Gln polymorphisms in non-small-cell lung cancer (NSCLC) is much higher than in normal controls.^[18]^ Another study suggested that, although no base mutation had been found in both NSCLC cancer cell lines and NSCLC cancer tissues, the frequency of SNPs at codon 302 tended to be higher in NSCLC patients compared to healthy volunteers.^[19]^ It has also been reported that genetic polymorphism of some *RAD18* loci is correlated with the prognosis of cancer patients^[20]^ and the side effects of platinum-chemotherapy.^[21]^

Cervical cancer remains a major cause of female mortality worldwide, particularly in developing countries that have limited screening programs.^[22]^ Only a small fraction (~1%) of women with cervical human papillomavirus (HPV) infection develop cervical neoplasia,^[23]^ but the factors determining the risk of progression are incompletely understood. The host genetic variation is a major determinant of the likelihood of cervical neoplasia in HPV-affected women. Further research is needed such as the potential for genetic risk score analysis in combination with other measures to identify subsets of women at particularly high risk of cervical neoplasia.^[24,25]^

To date, there is no literature involving genetic variations of the *RAD18* gene and susceptibility to cervical cancer. We designed a case-control study based on a large sample population, selecting 6 SNP loci from *RAD18* and detecting their distribution in the peripheral blood cell genomes of 650 cases of CIN III, 580 cases of cervical squamous cell carcinoma (CSCC), and 1320 normal healthy controls. We also investigated the relationship between different SNP genotypes and susceptibility to CSCC and CIN III, and conducted correlation analyses on corresponding clinical parameters related to prognosis, with the aim of better understanding the role of specific SNP genotypes in the carcinogenic process of CSCC.

## 2. Methods

### 2.1. Subjects

We conducted a study looking at the role of selected SNPs in CSCC and its precursor lesion CIN III. In total, 580 CSCC patients and 650 CIN III patients from the Women’s Hospital, School of Medicine, Zhejiang University China were investigated and diagnoses were confirmed by 2 pathologists. In total, 1320 healthy volunteers were enrolled as controls in this study. The inclusion criteria for the normal healthy controls were as follows: no history of tumors, no cervical cytological finding, no endometriosis, and no immune-related disease. Among CSCC, the expression of the *RAD18* protein was detected by immunohistochemistry on paraffin sections from 187 CSCC patient samples. Our research was approved by the Medical Ethical Committee of the Women’s Hospital, School of Medicine, Zhejiang University (No. 2004002). All patients signed informed consent for this molecular research.

The clinical and pathohistological characteristics examined were: age, tumor family history, FIGO stage, tumor size, differentiation grade, lymph node metastasis, vascular involvement, stromal invasion, vaginal wall extension, parametrial extension and endometrial extension. The above data were obtained from the records of the archives of the Women’s Hospital, School of Medicine, Zhejiang University.

### 2.2. Specimen preparation and genomic DNA extraction

Before the patients began their treatment, 2 mL of peripheral blood was collected in EDTA-anticoagulant tubes. Genomic DNA was extracted from the blood, using a whole blood gDNA extraction kit (Sangon Bio Co., Shanghai, China), following the manufacturer’s instructions. DNA quantification and quality were assessed using a NanoDrop 2000 (Thermo Fisher, Wilmington). Genomic DNA was dissolved in deionized water and frozen until use.

### 2.3. SNP pick-up and genetic analysis

A total of 6 tag-SNPs were picked up from a SNP library (https://www.ncbi.nlm.nih.gov/snp/). We utilized the filter option (filters activated: SNP missense, 5’-UTR, 3’-UTR, minor allele frequency [MAF] from 0.05 to 0.5) to obtain 6 effective SNPs in the *RAD18* gene (rs373572[A/G], rs615967[A/G], rs193920[A/C/T], rs250403[A/G], rs250404[C/T] and rs34927291[A/C/T]). One of these 6 SNPs was missensed in the coding region, 2 in the 5’-UTR region and 3 in the 3’-UTR region.

Based on the work of Kisaki et al,^[26]^ we designed allele-specific primers to detect specific nucleotides at each SNP site, then amplified them by PCR, and detected the products by agarose gel electrophoresis, and the different positive electrophoretic bands were used to determine the identity of nucleotide present. This method of detecting genetic variations in nucleotides is a modified allele-specific primer extension reaction. To prevent mismatched primer extensions, an artificial mismatched base was introduced at the second position from the 3’ terminal of the forward primer. The specific allele-specific primers and PCR product length are shown in Table S1 (Supplemental Digital Content, https://links.lww.com/MD/P735).

PCR was conducted in a total volume of 20 µL and contained 20 ng DNA, 5.0 pmol forward and reverse primers, 0.25 mM dNTP and 1.0 U of Taq DNA polymerase (TAKARA Co., Dalian, China). Thermal cycling was carried out using a PCR Thermal Cycler S1000 (BIO-RAD) and programmed as follows: 10 minutes at 94°C, followed by 35 cycles at 94°C for 30 seconds, 56−60°C for 30 seconds, 72°C for 30 seconds, and a final elongation at 72°C was performed for 5 minutes. Then, electrophoresis was conducted using a 2% agarose gel, and the products were stained with ethidium bromide. All experimental results were validated by 2 technicians under double-blind conditions.

### 2.4. Immunohistochemistry

The sections were firstly incubated with Rabbit anti-RAD18 Polyclonal antibody (1:200, 18333-1-AP, Proteintech), and then incubated with Dako Envision^TM^ Peroxidase (Dako Diagnostica, Hamburg, Germany), and visualized with 3,3’-diaminobenzidine tetrahydrochloride (Dako). All slides were counterstained with hematoxylin.

Immunohistochemical results were scored as follows: 0: <5% positive cells; 1: 5% to 25% positive cells; 2: 26% to 75% positive cells; 3: more than 76% positive cells. Stain intensity was scored as follows: 0, no staining; 1: faint-yellow; 2: brown-yellow; 3: dark-brown. The expression level (sum of the 2 scores) was finally defined as follows: (0), + (1–2), ++ (3–4), +++ (5–6). All the evaluations were made by 2 independent pathologists, unaware of the clinical data.

### 2.5. Statistical analyses

We compared the allele frequencies of the SNPs in the *RAD18* gene between the healthy control group and the patient groups with CIN III or CSCC. The distribution of the *RAD18* SNPs genotype in all of the patients and the healthy controls was tested for adherence to the Hardy-Weinberg equilibrium. The binary logistic regression analysis was used to obtain odds ratios (ORs), 95% confidence intervals (CIs), and *P* values.

The normal control group acted as the reference. The OR and 95% CI were both adjusted for age, sex, and smoking status using an unconditional logistic regression model. FDR adjusted p values were corrected using the Benjamin–Hochberg method for multiple testing corrections. The relationship between the genotype distribution frequency and the clinicopathological parameters was examined using the Kruskal–Wallis *H* test. Multinomial regression analysis was performed among the different groups for different genotypes. The immunohistochemistry looking at protein expression was assessed using the Kruskal–Wallis *H* test and the Mann–Whitney *U* test A *P*-value of ≤.05 was considered to be statistically significant. The statistical analyses were performed using SPSS (IBM Corp., Armonk) software (Version 18.0 for Windows).

## 3. Results

### 3.1. Association between genetic polymorphisms of *RAD18* with the risk of CIN III or CSCC

Table 1 shows the genotypes and allele frequencies for the 6 genetic polymorphism loci of *RAD18* (rs373572, rs615967, rs193920, rs250403, rs250404, rs34927291). All genotype frequency distributions met the requirements of the Hardy–Weinberg equilibrium (Table S2, Supplemental Digital Content, https://links.lww.com/MD/P735).

**Table 1:** Association between RAD18 genetic variants and the risk of CIN III and CSCCs.

Genotypes	Controls (N = 1320)		CIN III (N = 650)		Adjusted OR* (95% CI)	*P*	CSCCs (N = 580)		Adjusted OR* (95% CI)	*P*
	N	%	N	%			N	%		
rs373572										
AA	562	42.6	282	43.4	1.00 (ref)		244	42.1	1.00 (ref)	
AG	588	44.5	274	42.2	0.929 (0.758–1.137)	.474	259	44.7	1.015 (0.823–1.251)	.893
GG	170	12.9	94	14.5	1.102 (0.825–1.472)	.511	77	13.3	1.043 (0.766–1.420)	.788
AG + GG	758	57.4	368	56.6	0.968 (0.800–1.170)	.733	336	57.9	1.021 (0.838–1.244)	.837
Allelic frequency										
Allele A	1712	64.8	838	64.5	1.00 (ref)		747	64.4	1.00 (ref)	
Allele G	928	35.2	462	35.5	1.017 (0.885–1.169)	.811	413	35.6	1.020 (0.883–1.178)	.788
rs615967										
AA	477	36.1	202	31.1	1.00 (ref)		154	26.6	1.00 (ref)	
AG	624	47.3	321	49.4	1.215 (0.982–1.502)	.073	288	49.7	**1.430 (1.137–1.798**)	**.002**
GG	219	16.6	127	19.5	**1.369 (1.042–1.800**)	**.024**	138	23.8	**1.952 (1.475–2.582**)	**.000**
AG + GG	843	63.9	448	68.9	**1.255 (1.027–1.534**)	**.026**	426	73.4	**1.565 (1.261–1.942**)	**.000**
Allelic frequency										
Allele A	1578	59.8	725	55.8	1.00 (ref)		596	51.4	1.00 (ref)	
Allele G	1062	40.2	575	44.2	**1.178 (1.030–1.348**)	**.017**	564	48.6	**1.406 (1.224–1.616**)	**.000**
rs193920										
CC	678	51.4	323	49.7	1.00 (ref)		303	52.2	1.00 (ref)	
CT	532	40.3	259	39.8	1.022 (0.837–1.247)	.831	223	38.4	0.938 (0.763–1.154)	.544
TT	110	8.3	68	10.5	1.298 (0.933–1.805)	.122	54	9.3	1.098 (0.772–1.563)	.602
CT + TT	642	48.6	327	50.3	1.069 (0.886–1.290)	.485	277	47.8	0.965 (0.794–1.174)	.724
Allelic frequency										
Allele C	1888	71.5	905	69.6	1.00 (ref)		829	71.5	1.00 (ref)	
Allele T	752	28.5	395	30.4	1.096 (0.948–1.267)	.217	331	28.5	1.002 (0.860–1.168)	.975
rs250403										
AA	804	60.9	355	54.6	1.00 (ref)		262	45.2	1.00 (ref)	
AG	437	33.1	214	32.9	1.109 (0.903–1.362)	.324	187	32.2	**1.313 (1.053–1.638**)	**.016**
GG	79	6.0	81	12.5	**2.322 (1.663–3.243**)	**.000**	131	22.6	**5.089 (3.724–6.952**)	**.000**
AG + GG	516	39.1	295	45.4	**1.295 (1.071–1.566**)	**.008**	318	54.8	**1.891 (1.552–2.304**)	**.000**
Allelic frequency										
Allele A	2045	77.5	924	71.1	1.00 (ref)		711	61.3	1.00 (ref)	
Allele G	595	22.5	376	28.9	**1.399 (1.203–1.626**)	**.000**	449	38.7	**2.170 (1.869–2.520**)	**.000**
rs250404										
TT	364	27.6	168	25.8	1.00 (ref)		166	28.6	1.00 (ref)	
TC	658	49.8	315	48.5	1.037 (0.827–1.301)	.752	271	46.7	0.903 (0.716–1.139)	.389
CC	298	22.6	167	25.7	1.214 (0.933–1.580)	.148	143	24.7	1.052 (0.802–1.380)	.713
TC + CC	956	72.4	482	74.2	1.092 (0.883–1.352)	.416	414	71.4	0.950 (0.765–1.179)	.640
Allelic frequency										
Allele T	1386	52.5	651	50.1	1.00 (ref)		603	52.0	1.00 (ref)	
Allele C	1254	47.5	649	49.9	1.102 (0.965–1.258)	.152	557	48.0	1.021 (0.889–1.172)	.769
rs34927291										
CC	1147	86.9	567	87.2	1.00 (ref)		506	87.2	1.00 (ref)	
CT	163	12.3	79	12.2	0.980 (0.736–1.306)	.893	69	11.9	0.960 (0.711–1.296)	.788
TT	10	0.8	4	0.6	0.809 (0.253–2.591)	.721	5	0.9	1.133 (0.385–3.333)	.820
CT + TT	173	13.1	83	12.8	0.971 (0.733–1.285)	.834	74	12.8	0.970 (0.724–1.298)	.836
Allelic frequency										
Allele C	2457	93.1	1213	93.3	1.00 (ref)		1081	93.2	1.00 (ref)	
Allele T	183	6.9	87	6.7	0.963 (0.739–1.254)	.780	79	6.8	0.981 (0.746–1.290)	.892

* All *P*-values are adjusted for age, number of sexual partners, age at first intercourse, parities (including full-term pregnancy and abortion at or after 28 wk) and age at first full-term pregnancy.

The frequency of the genotype distribution found that 4 genetic polymorphisms (rs373572, rs193920, rs250404, rs34927291) were not associated with the risk of CIN III or CSCC. The AA, AG, and GG genotype frequencies of *RAD18* rs615967 were 36.1%, 47.3%, and 16.6% in normal controls; 31.1%, 49.4%, and 19.5% in the CIN III group and 26.6%, 49.7%, and 23.8% in the CSCC group, respectively. Patients with the rs615967 GG genotype had a significantly higher risk of CIN III [OR = 1.369; 95% CI: 1.042−1.800] and CSCC [OR = 1.952; 95% CI: 1.475−2.582). We also found that the frequency of G alleles at the rs615967 in the CIN III group (575/1300, 44.2%) and CSCC group (564/1160, 48.6%) were significantly higher than those in normal controls (1062/2640, 40.2%). The OR of the G allele in the CIN III group was 1.178 (95% CI: 1.030−1.348) and 1.406 (95% CI: 1.224−1.616) in the CSCC group. Carriers of the G allele (AG + GG) at rs615967 were associated with a higher risk of CIN III (OR = 1.255; 95% CI: 1.027−1.534) and CSCC (OR = 1.565; 95% CI: 1.261−1.942).

The AA, AG, and GG genotype frequencies of *RAD18* rs250403 were 60.9%, 33.1%, and 6.0% in the controls; 54.6%, 32.9%, and 12.5% in the CIN III group and 45.2%, 32.2%, and 22.6% in the CSCC group, respectively. Patients carrying the heterozygote AG genotype rs250403 also had a significantly elevated risk of CSCC (OR = 1.313; 95% CI: 1.053−1.638). The G allele frequencies of rs250403 in the CIN III (376/1300, 28.9%) and CSCC groups (449/1160, 38.7%) were higher than those in normal controls (595/2640, 22.5%). The G allele was associated with a higher risk of both CIN III (OR = 1.399; 95% CI: 1.203−1.626) and CSCC (OR = 2.170; 95% CI: 1.869−2.520), respectively. Carriers of the G allele (AG + GG) at rs250403 were associated with a higher risk of CIN III (OR = 1.295; 95% CI: 1.071−1.566) and CSCC (OR = 1.891; 95% CI: 1.552−2.304).

### 3.2. The relationship between *RAD18* rs250403 and rs615967 gene polymorphisms and sexual and reproductive history in the CIN III and CSCC groups

Stratified analysis was conducted to analyze the association between the *RAD18* rs250403 and rs615967 genotypes and age, number of sexual partners, age at first intercourse, number of parities, age at first parity, and HR-HPV infection status. There was no enrichment between CIN III and CSCC and *RAD18* rs250403, rs615967 genetic polymorphism (Tables 2 and 3).

**Table 2:** Association between RAD18 rs250403 polymorphisms and the risk for CIN III and CSCCs stratified by the sexual, reproductive history.

High risk exposure	Normal healthy controls						χ^2^	*P*	CIN III						χ^2^	*P*	CSCCs						χ^2^	*P*
	AA		AG		GG				AA		AG		GG				AA		AG		GG			
	N	%	N	%	N	%			N	%	N	%	N	%			N	%	N	%	N	%		
Age																								
≤40	413	61.5	219	32.6	39	5.8	0.249	.618	231	54.7	137	32.5	54	12.8	0.001	.977	104	44.4	74	31.6	56	23.9	0.231	.631
>40	391	60.2	218	33.6	40	6.2			124	54.4	77	33.8	27	11.8			158	45.7	113	32.7	75	21.7		
Number of sexual partners																								
≤1	649	61.3	348	32.9	61	5.8	0.526	.468	281	54.5	173	33.5	62	12.0	0.004	.951	201	44.6	151	33.5	99	22.0	0.010	.920
>1	155	59.2	89	34.0	18	6.9			74	55.2	41	30.6	19	14.2			61	47.3	36	27.9	32	24.8		
Age at the first intercourse																								
≤20	231	58.9	129	32.9	32	8.2	1.669	.196	122	57.0	71	33.2	21	9.8	1.300	.254	87	46.5	63	33.7	37	19.8	0.638	.424
>20	573	61.7	308	33.2	47	5.1			233	53.4	143	32.8	60	13.8			175	44.5	124	31.6	94	23.9		
Number of parities																								
≤3	368	61.2	201	33.4	32	5.3	0.141	.707	136	52.5	89	34.4	34	13.1	0.743	.389	87	47.8	49	26.9	46	25.3	0.029	.865
>3	436	60.6	236	32.8	47	6.5			219	56.0	125	32.0	47	12.0			175	44.0	138	34.7	85	21.4		
Age at the first parity																								
≤22	166	64.8	78	30.5	12	4.7	2.284	.131	77	52.0	47	31.8	24	16.2	1.132	.287	53	43.4	37	30.3	32	26.2	0.597	.440
>22	638	60.0	359	33.7	67	6.3			278	55.4	167	33.3	57	11.4			209	45.6	150	32.8	99	21.6		
HR-HPV infection status																								
Positive	131	60.1	73	33.5	14	6.4	0.203	.653	296	53.2	188	33.8	72	12.9	0.129	.720	132	43.7	94	31.1	76	25.2	0.074	.785
Negative	301	58.9	168	32.9	42	8.2			28	57.1	14	28.6	7	14.3			12	46.2	8	30.8	6	23.1		

Stratified analysis were applied by the Kruskale–Wallis *H* test. A *P* value <.05 was considered significant.

**Table 3:** Association between RAD18 rs615967 polymorphisms and the risk for CIN III and CSCCs stratified by the sexual, reproductive history.

High risk exposure	Normal healthy controls						χ^2^	*P*	CIN III						χ2	*P*	CSCCs						χ^2^	*P*
	AA		AG		GG				AA		AG		GG				AA		AG		GG			
	N	%	N	%	N	%			N	%	N	%	N	%			N	%	N	%	N	%		
Age																								
≤40	241	35.9	314	46.8	116	17.3	0.192	.662	131	31.0	209	49.5	82	19.4	0.001	0.976	61	26.1	115	49.1	58	24.8	0.167	.682
>40	236	36.4	310	47.8	103	15.9			71	31.1	112	49.1	45	19.7			93	26.9	173	50.0	80	23.1		
Number of sexual partners																								
≤1	382	36.1	497	47.0	179	16.9	0.105	.746	161	31.2	255	49.4	100	19.4	0.039	0.843	119	26.4	223	49.4	109	24.2	0.116	.733
>1	95	36.3	127	48.5	40	15.3			41	30.6	66	49.3	27	20.1			35	27.1	65	50.4	29	22.5		
Age at the first intercourse																								
≤20	142	36.2	186	47.4	64	16.3	0.012	.914	68	31.8	106	49.5	40	18.7	0.149	0.699	48	25.7	91	48.7	48	25.7	0.411	.521
>20	335	36.1	438	47.2	155	16.7			134	30.7	215	49.3	87	20.0			106	27.0	197	50.1	90	22.9		
Number of parities																								
≤3	218	36.3	287	47.8	96	16.0	0.101	.750	81	31.3	126	48.6	52	20.1	0.007	0.934	49	26.9	88	48.4	45	24.7	0.015	.902
>3	259	36.0	337	46.9	123	17.1			121	30.9	195	49.9	75	19.2			105	26.4	200	50.3	93	23.4		
Age at the first parity																								
≤22	93	36.3	123	48.0	40	15.6	0.068	.794	48	32.4	69	46.6	31	20.9	0.001	0.975	31	25.4	64	52.5	27	22.1	0.007	.935
>22	384	36.1	501	47.1	179	16.8			154	30.7	252	50.2	96	19.1			123	26.9	224	48.9	111	24.2		
HR-HPV infection status																								
Positive	79	36.2	104	47.7	35	16.1	0.031	.860	174	31.3	256	46.0	126	22.7	0.002	0.962	71	23.5	138	45.7	93	30.8	0.235	.628
Negative	189	37.0	241	47.2	81	15.9			15	30.6	23	46.9	11	22.4			7	26.9	12	46.2	7	26.9		

Stratified analysis were applied by the Kruskale–Wallis *H* test. A *P* value <.05 was considered significant.

### 3.3. Association between *RAD18* rs250403 and rs615967 polymorphism and the risk for cervical carcinoma stratified by clinical pathological characteristics (mainly prognostic factors)

Correlations with RAD18 rs250403 and rs615967 polymorphisms and the clinicopathological characteristics of CSCC are shown in Tables 4 and 5.

**Table 4:** Association between RAD18 rs250403 polymorphism and the risk for cervical carcinoma stratified by clinical pathological characteristics.

Clinical pathological characteristics	RAD18 rs250403						χ^2^	*P*
	AA		AG		GG			
	N	%	N	%	N	%		
Age								
≤40	107	45.7	71	30.3	56	23.9	0.013	.909
>40	155	44.8	116	33.5	75	21.7		
Tumor family history								
Negative	243	45.4	173	32.3	119	22.2	0.346	.556
Positive	19	42.2	14	31.1	12	26.7		
FIGO stage								
I	221	44.9	167	33.9	104	21.1	0.402	.526
II	41	46.6	20	22.7	27	30.7		
Tumor size								
<4 cm	212	43.5	163	33.5	112	23.0	2.359	.125
≥4 cm	50	53.8	24	25.8	19	20.4		
Differentiation grade								
Grade I–II	231	46.5	169	34.0	97	19.5	**8.750**	**.003**
Grade III	31	37.3	18	21.7	34	41.0		
Lymph node metastasis								
Negative	241	46.5	166	32.0	111	21.4	**4.758**	**.029**
Positive	21	33.9	21	33.9	20	32.3		
Vascular involvement								
Negative	226	45.8	168	34.1	99	20.1	**4.082**	**.043**
Positive	36	41.4	19	21.8	32	36.8		
Stromal invasion								
Negative	188	46.2	133	32.7	86	21.1	1.193	.275
Positive	74	42.8	54	31.2	45	26.0		
Vaginal wall extension								
Negative	199	43.5	151	33.0	107	23.4	2.196	.138
Positive	63	51.2	36	29.3	24	19.5		
Parametrail extension								
Negative	237	45.5	165	31.7	119	22.8	0.020	.888
Positive	25	42.4	22	37.3	12	20.3		
Endometrial extension								
Negative	244	45.8	171	32.1	118	22.1	1.166	.28
Positive	18	38.3	16	34.0	13	27.7		

Stratified analysis were applied by the Kruskale–Wallis *H* test. A *P* value <.05 was considered significant.

**Table 5:** Association between RAD18 rs615967 polymorphism and the risk for cervical carcinoma stratified by clinical pathological characteristics.

Clinical pathological characteristics	RAD18 rs615967						χ^2^	*P*
	AA		AG		GG			
	N	%	N	%	N	%		
Age								
≤40	64	27.4	119	50.9	51	21.8	0.599	.439
>40	90	26.0	169	48.8	87	25.1		
Tumor family history								
Negative	147	27.5	267	49.9	121	22.6	**6.012**	**.014**
Positive	7	15.6	21	46.7	17	37.8		
FIGO stage								
I	132	26.8	244	49.6	116	23.6	0.157	.692
II	22	25.0	44	50.0	22	25.0		
Tumor size								
<4 cm	131	26.9	243	49.9	113	23.2	0.524	.469
≥4 cm	23	24.7	45	48.4	25	26.9		
Differentiation grade								
Grade I–II	141	28.4	249	50.1	107	21.5	**11.435**	**.001**
Grade III	13	15.7	39	47.0	31	37.3		
Lymph node metastasis								
Negative	144	27.8	258	49.8	116	22.4	**6.719**	**.010**
Positive	10	16.1	30	48.4	22	35.5		
Vascular involvement								
Negative	132	26.8	247	50.1	114	23.1	0.510	.475
Positive	22	25.3	41	47.1	24	27.6		
Stromal invasion								
<2/3	107	26.3	207	50.9	93	22.9	0.118	.732
≥2/3	47	27.2	81	46.8	45	26.0		
Vaginal wall extension								
Negative	123	26.9	228	49.9	106	23.2	0.391	.532
Positive	31	25.2	60	48.8	32	26.0		
Parametrail extension								
Negative	138	26.5	261	50.1	122	23.4	0.094	.759
Positive	16	27.1	27	45.8	16	27.1		
Endometrial extension								
Negative	142	26.6	265	49.7	126	23.6	0.076	.782
Positive	12	25.5	23	48.9	12	25.5		

Stratified analysis were applied by the Kruskale–Wallis *H* test. A *P* value <.05 was considered significant.

Stratified analysis was performed based on age, tumor family history, FIGO stage, tumor size, differentiation grade, lymph node metastasis, vascular involvement, stromal invasion, vaginal wall extension, parametrial extension, and endometrial extension. We found that the polymorphism of *RAD18* rs250403 was significantly correlated with differentiation grade (χ^2^ = 8.750, *P* = .003), lymph node metastasis (χ^2^ = 4.758, *P* = .029), and vascular involvement (χ^2^ = 4.082, *P* = .043), while *RAD18* rs615967 was significantly correlated with tumor family history (χ^2^ = 6.012, *P* = .014), differentiation grade (χ^2^ = 11.435, *P* = .001), and lymph node metastasis (χ^2^ = 6.719, *P* = .010).

### 3.4. Association between *RAD18* rs250403 (A/G) and rs615967 (A/G) haplotypes and the risk of CIN III and CSCC

We analyzed the linkage disequilibrium between the genotype frequencies of rs250403 (A/G) and rs615967 (A/G), as these 2 genetic polymorphisms were significantly associated with the risk of CIN III and CSCC.

As shown in Table 6, when compared with the reference haplotype (AA-AA), the haplotypes of AG-GG (OR = 1.827; 95% CI: 1.176−2.840), GG-AA (OR = 2.033; 95% CI: 1.100−3.760), GG-AG (OR = 2.436; 95% CI: 1.410−4.210), and GG-GG (OR = 3.433; 95% CI: 1.900−6.202) were significantly associated with an increased risk of CIN III. for CSCCs, a higher risk was detected with AG-AG (OR = 1.674; 95% CI: 1.182−2.370), AG-GG (OR = 2.789; 95% CI: 1.771−4.393), GG-AA (OR = 4.529; 95% CI: 2.549−8.047), GG-AG (OR = 6.647; 95% CI: 4.011−11.015), and GG-GG (OR = 7.192; 95% CI: 4.061−12.736). These data indicated that the linkage mode of rs250403 (A/G) and *RAD18* rs615967 (A/G) was associated with an elevated risk for CIN III and CSCC and the riskiest genetic linkage mode was GG-GG. Therefore, these specific linkage patterns were associated with a higher risk of CIN III or CSCC. The haplotypes of AG-GG, GG-AA, GG-AG, and GG-GG at rs250403 and rs615967 in the *RAD18* gene may act as a genetic predictive biomarker for susceptibility of CIN III and/or CSCC.

**Table 6:** RAD18 haplotype of rs250403(A/G) and rs615967(A/G) and the risk of CIN III and CSCCs.

RAD18 Genotypes*	Normal healthy controls		CIN III		Adjusted OR† (95% CI)	*P*	CSCCs		Adjusted OR† (95% CI)	*P*
	1320		650				580			
	n	%	n	%			n	%		
AA-AA	287	21.7	114	17.5	1.00 (ref)		78	13.4	1.00 (ref)	
AA-AG	382	28.9	189	29.1	1.246 (0.943–1.645)	.122	136	23.4	1.310 (0.953–1.800)	.096
AA-GG	135	10.2	52	8.0	0.970 (0.659–1.427)	.876	48	8.3	1.308 (0.865–1.979)	.203
AG-AA	164	12.4	67	10.3	1.029 (0.719–1.471)	.878	44	7.6	0.987 (0.651–1.497)	.952
AG-AG	211	16.0	102	15.7	1.217 (0.883–1.677)	.230	96	16.6	**1.674 (1.182–2.370**)	**.004**
AG-GG	62	4.7	45	6.9	**1.827 (1.176–2.840**)	**.007**	47	8.1	**2.789 (1.771–4.393**)	**.000**
GG-AA	26	2.0	21	3.2	**2.033 (1.100–3.760**)	**.024**	32	5.5	**4.529 (2.549–8.047**)	**.000**
GG-AG	31	2.3	30	4.6	**2.436 (1.410–4.210**)	**.001**	56	9.7	**6.647 (4.011–11.015**)	**.000**
GG-GG	22	1.7	30	4.6	**3.433 (1.900–6.202**)	**.000**	43	7.4	**7.192 (4.061–12.7360**	**.000**

* Genotypes are composed of 2 polymorphic sites: rs250403 (A/G), rs615967 (A/G).

† All *P*-values are adjusted for age, number of sexual partners, age at first intercourse, parities (including full-term pregnancy and abortion at or after 28 wk) and age at first full-term pregnancy.

### 3.5. Protein expression of RAD18 in CSCC with different rs250403 (A/G) or rs615967 (A/G) genotypes

**Figure 1.**
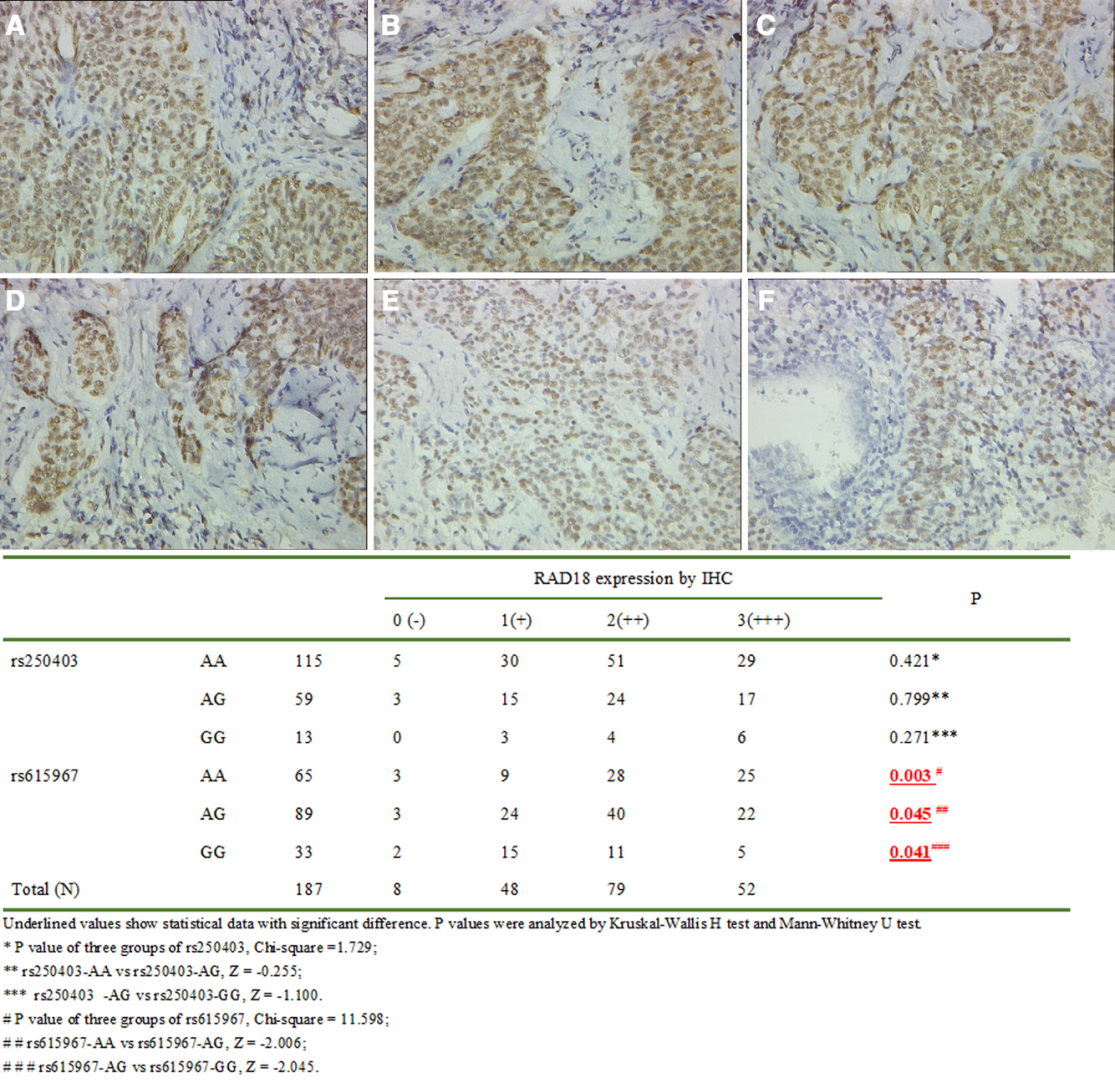
RAD18 expression in CSCC with different genetic polymorphisms as determined by immunohistochemistry (40× objective, magnified). (A) rs250403-AA; (B) rs250403-AG; (C) rs250403-GG; (D) rs615967-AA; (E) rs615967-AG; (F) rs615967-GG. The distinct brown coloration is mainly located in the nucleus of the positive cells.

As shown in Figure 1A to C, among the 187 cases of CSCC, the frequencies of AA, AG, and GG genotypes of rs250403 were 115 (61.5%), 59 (31.6%), and 13 (6.9%), respectively. When the rs250403 (AA) was used as the control group, there was no significant difference in the expression of RAD18 protein when compared with the different genotype groups (χ^2^ = 1.729, *P* = .421).

The frequencies of AA, AG and GG genotypes of rs615967 in the 187 CSCC patients were 65 (34.8%), 89 (47.6%), and 33 (17.6%). When rs615967 (AA) was used as the control, the expression of the RAD18 protein with the rs615967 (AG) genotype decreased by approximately 15%, and the rs615967 (GG) genotype decreased by approximately 33.9%. The expression of the RAD18 protein in patients with rs615967 (AG) and rs615967 (GG) were significantly lower than in patients with rs250403 (AA) (χ^2^ = 11.598, *P* = .003) (Fig. 1D–F).

## 4. Discussion

The development of cervical cancer is strongly associated with genital infection from oncogenic types of HPV. However, the majority of women infected with HPV never develop cancer. Pedigree studies show that cervical cancer has a significant heritability factor and genetic predisposing factors may influence the likelihood of sensitivity to, or persistence of HPV infection, as well as the rate of tumor development.^[27]^ This suggests that genomic stability and genetic susceptibility play a critical role in the etiology of the genetic susceptibility of cervical cancer. Many studies, including 2 genome-wide association studies, have identified susceptibility loci and genetic variants in cervical cancer.^[28–30]^ Our previous studies have also found that 2 SNP loci in the *SMUG1* gene are significantly correlated with susceptibility to cervical cancer and HR-HPV infection, further supporting the important role of genomic genetic stability in cervical cancer.^[31]^

Here, we determined whether the polymorphism of 6 SNPs within the *RAD18* gene with MAF values of more than 5% was associated with the occurrence, progression, and prognostic risk of CIN III or CSCC. We found that the polymorphism of 4 SNPs (rs373572, rs193920, rs250404, rs34927291) did not differ in distribution among CIN III, CSCC or and healthy control groups, while there were significant differences in genotype distribution between the rs615967 (A/G) and rs250403 (A/G) loci. Furthermore, the GG homozygosity at rs615967 or carrying of the G allele (AG + GG) increased the risk of developing CIN III or CSCC. The GG homozygotes at rs250403 and those carrying the G allele (AG + GG) also have the same distribution and higher risk, especially the GG homozygotes at rs250403 and have an OR value of 5.089 in CSCC.

We compared the *RAD18* rs250403 (A/G) and rs615967 (A/G) haplotypes with the reference genotype AA-AA and found that haplotypes AG-GG, GG-AA, GG-AG, and GG-GG were significantly associated with an increased risk of CIN III. In addition, in CSCC, the risk of haplotypes possessing AG-AG, AG-GG, GG-AA, GG-AG, and GG-GG was much higher. Especially when both loci exhibited a G allele, the impact on disease susceptibility was much greater than when these 2 loci were analyzed separately. When both loci exhibited haplotypes of the GG homozygous type (GG-GG), the OR values for CIN III and CSCC were 3.433 and 7.192, respectively. A higher OR value, combined with statistical significance, indicated a synergistic effect between the rs250403 and rs615967 genetic polymorphisms in the *RAD18* gene. This synergistic effect may promote the development of CIN III, ultimately leading to cervical cancer.

As is well-known, persistent infection with high-risk HPV is a prerequisite for the occurrence of cervical cancer. Approximately 99.7% of cervical cancer cases are caused by persistent genital high-risk human papillomavirus infection.^[32]^ It is interesting that although the rs250403 and rs615967 polymorphisms are significantly associated with the occurrence of CIN III and CSCC, when the stratified analysis was conducted on high-risk HPV infection, patient age, age of first sexual intercourse, frequency of childbirth, and age of first childbirth, (which are considered to be associated with an increased risk of cervical cancer),^[33,34]^ we found that these characteristics were not associated with the polymorphisms of the 2 SNPs suggesting that their pathogenic role was not through increased susceptibility to high-risk HPV, but through the pathogenic process after HPV infection.

In 2007, Kanzaki et al detected the *RAD18* SNP (Arg302Gln) gene polymorphism in 100 colorectal cancer patients and 200 healthy controls in the Japanese population. They found a significant difference in genotype frequency between the control and the patient groups. In the control group, the frequencies of the Arg/Arg, Arg/Gln, and Gln/Gln genotypes were 43.0%, 45.5%, and 11.5%, respectively, while in colorectal cancer patients, they were 32.0%, 50.0%, and 18.0%, respectively. Compared with the control group with the Arg/Arg genotype, colorectal cancer patients with homozygous Gln/Gln (A/A) genotype showed the most significant increase in risk (OR = 2.10), and the Gln allele enhances susceptibility to colorectal cancer development.^[17]^ Another study on 159 non-small cell lung cancer patients also showed a correlation between *RAD18*-Arg302Gln polymorphism and the risk of non-small cell lung cancer in humans. The frequency of Gln/Gln genotype in non-small cell lung cancer patients (20.7%) was significantly higher than that in the healthy control group (11.5%), and the Gln/Gln genotype was detected to increase risk in non-small cell lung cancer patients (OR = 2.63).^[18]^ The *RAD18* SNP (Arg302Gln) in these 2 studies was located in the coding region of the *RAD18* gene, but we are also very interested in the genetic variations in the noncoding region. Considering that over 90% of the associated genetic variations in the human genome are in the noncoding region of the genome, the noncoding region has the highest genome-wide association studies heritability (5-fold), and the genetic variations in these regions are crucial for understanding human phenotypic variations.^[35]^ Therefore, we selected 6 SNPs with MAF values of more than 5% in the noncoding region of the *RAD18* gene, all of which are located in the 5’-UTR promoter region or the 3’-UTR. Among them, rs615967, which is significantly associated with cervical cancer susceptibility, is located in the 5’-UTR promoter region and rs250403 is located in the 3’-UTR. These genetic variations in noncoding regions may affect gene function through the regulation of transcription, post-transcriptional modifications, and translational processes. Therefore, further functional studies are needed to elucidate their regulatory mechanisms.

Considering that *RAD18* rs615967 (A/G) is located in the 5‘-UTR promoter region, we believe that genetic variations may affect gene expression. Therefore, we measured the expression of RAD18 protein in the pathological tissues of 187 CSCC patients. We found that RAD18 protein was significantly reduced in the patients with the rs615967-AG and GG genotypes, indicating that the effect of *RAD18* SNP (rs615967) on cervical cancer susceptibility may be due to changes in *RAD18* expression, leading to a decrease in the ability to repair damaged genomes, resulting in genomic instability and tumorigenesis.We also found that although rs250403 (A/G) located in the 3’-UTR has a higher susceptibility risk for cervical cancer (rs250403 GG with an OR = 5.089), the different genotypes of rs250403 (A/G) did not lead to differences in protein expression. Therefore, we speculate that the rs250403 (A/G) SNP may change the spatial structure of protein functional domains by altering non synonymous changes in amino acid sequences, thereby affecting the level of DNA repair activity in cells, inducing genomic instability, and ultimately leading to cervical cancer.

Furthermore, we analyzed the correlation between the polymorphisms of rs250403 and rs615967 and some clinical pathological features related to the prognosis of cervical cancer. We found that the polymorphisms of rs615967 and rs250403 were significantly correlated with lymph node metastasis and tumor differentiation. In addition, rs615967 was also associated with tumor family history and rs250403 was correlated with the degree of vascular involvement. Our findings are consistent with the results of other studies. There is a significant correlation between *RAD18* SNP (Arg302Gln) gene polymorphisms and clinicopathological parameters in colorectal cancer, especially in terms of the degree of differentiation (OR = 7.00) and lymph node metastasis (OR = 3.71). In patients with elevated differentiation and lymph node metastasis (N1), the detection frequency of the Gln allele was higher.^[17]^ Another study also found that in patients with colorectal cancer, the disease-free survival (DFS) in GG genotype patients with the *RAD18* SNP of rs373572 was low. Compared with AG or AA genotype patients, the 1-year, 3-year, and 5-year DFS in patients with the GG genotype and the rs373572 *RAD18* SNP were 86.7%, 53.3%, and 45.7%, respectively, while the 1-year, 3-year, and 5-year DFS in AG/AA genotype patients were 94.9%, 78.9%, and 74.2%, respectively. Especially in stage I colorectal cancer patients, the GG genotype is seen more commonly in patients with recurrent disease, making it a potential negative prognostic factor for early colorectal cancer diagnosis.^[20]^

To summarize, these results indicated that the polymorphisms of *RAD18* rs250403 and rs615967 were associated with disease susceptibility, disease progression, and prognosis in CIN III and CSCC. Some specific high-risk haplotypes (AG-GG, GG-AA, GG-AG, and GG-GG) linked by rs250403 and rs615967 serve as genetic biomarkers for predicting susceptibility to CIN III and CSCC. This is the first report providing evidence for the association between the *RAD18* gene polymorphism and human cervical cancer risk.

## Acknowledgments

We thank International Science Editing (http://www.internationalscienceediting.com) for editing this manuscript.

## Author contributions

**Conceived and designed the experiments:** Feng Ye, Rui Zhang.

**Performed the experiments:** Rui Zhang, Jianping Kong, Yun Li, Hanzhi Wang, Qi Cheng, Caiyun Zhou, Minghua Yu.

**Analyzed the data:** Jianping Kong, Yitong Wang.

**Contributed reagents/materials/analysis tools:** Feng Ye, Hanzhi Wang, Qi Cheng.

**Wrote the paper:** Feng Ye, Rui Zhang.

**Conceptualization:** Feng Ye, Jianping Kong.

**Funding acquisition:** Feng Ye.

**Investigation:** Rui Zhang, Yun Li, Hanzhi Wang.

**Methodology:** Hanzhi Wang, Qi Cheng.

**Validation:** Hanzhi Wang. Caiyun Zhou, Minghua Yu.

**Writing – original draft:** Rui Zhang.

**Writing – review & editing:** Feng Ye.

## Supplementary Material


