# Supplementary material for: Risk of cervical squamous cell carcinoma associated with a single nucleotide polymorphism in the RAD18 gene in the Chinese population and its significance as a predictive biomarker
Source: Medicine (Baltimore). 2025 Aug 22;104(34):e44017. doi: 10.1097/MD.0000000000044017 (PMC12385099; doi:10.1097/MD.0000000000044017)
Supplement: Supplementary file 1 [file medi-104-e44017-s001.docx]

**Supplementary Tables:**

**Table S1:** The allele-specific forward, reverse primers and PCR product length

| **Gene Name** | **SNP No.** | **Forward primer** | | **Reverse primer** | **Product length** |
| --- | --- | --- | --- | --- | --- |
| **RAD18** | rs373572 **[A/G]** | For “**G**” | 5’-ACAGCTGCTGAAATAGTTGG-3’ | 5’-ACTAAGTGGAAATGTGTTAG-3’ | 197bp |
|  |  | For “**A**” | 5’-ACAGCTGCTGAAATAGTTGA-3’ |  |  |
|  | rs615967 **[A/G]** | For “**G**” | 5’-CTGACCCCCAGCATCCTCCG-3’ | 5’-AGCCACGCGGTCGGCTAGTG-3’ | 238bp |
|  |  | For “**A**” | 5’-CTGACCCCCAGCATCCTCCA-3’ |  |  |
|  | rs193920 **[A/C/T]** | For “**T**” | 5’-TGCCATTCTGAGCTGATGAT-3’ | 5’-ATTGAGCACTATCTGTTAGC-3’ | 271bp |
|  |  | For “**C**” | 5’-TGCCATTCTGAGCTGATGAC-3’ |  |  |
|  |  | For “**A**” | 5’-TGCCATTCTGAGCTGATGAA-3’ |  |  |
|  | rs250403 **[A/G]** | For “**G**” | 5’-ACACATCAGTCTTTACCAAG-3’ | 5’-TGCAGGTGAGCTGCTGGATG-3’ | 201bp |
|  |  | For “**A**” | 5’-ACACATCAGTCTTTACCAAA-3’ |  |  |
|  | rs250404 **[C/T]** | For “**C**” | 5’-AAAGCTGATGTGTTTGGCC-3’ | 5’-CTGTAACAATGAATCCAAGC-3’ | 286bp |
|  |  | For “**T**” | 5’-AAAGCTGATGTGTTTGGCT-3’ |  |  |
|  | rs34927291 **[A/C/T]** | For “**T**” | 5’-GATGCTGGGGGTCAGCCAGT-3’ | 5’-GGCGCATGCGCAGTACAAGC-3’ | 176bp |
|  |  | For “**C**” | 5’-GATGCTGGGGGTCAGCCAGC-3’ |  |  |
|  |  | For “**A**” | 5’-GATGCTGGGGGTCAGCCAGA-3’ |  |  |

**Supplementary Tables:**

**Table S2:** Hardy-Weinberg Equilibrium test for the alleles of SNPs in normal healthy controls

| Genotypes | | | Control ( n=1320) | |
| --- | --- | --- | --- | --- |
|  |  |  |  |  |
|  |  |  | n | *P* |
|  | **rs373572** | |  | 0.405 |
|  |  | AA | 562 |  |
|  |  | AG | 588 |  |
|  |  | GG | 170 |  |
|  | **rs615967** | |  | 0.537 |
|  |  | AA | 477 |  |
|  |  | AG | 624 |  |
|  |  | GG | 219 |  |
|  | **rs193920** | |  | 0.695 |
|  |  | CC | 678 |  |
|  |  | CT | 532 |  |
|  |  | TT | 110 |  |
|  | **rs250403** | |  | 0.060 |
|  |  | AA | 804 |  |
|  |  | AG | 437 |  |
|  |  | GG | 79 |  |
|  | **rs250404** | |  | 0.985 |
|  |  | TT | 364 |  |
|  |  | TC | 658 |  |
|  |  | CC | 298 |  |
|  | **rs34927291** | |  | 0.119 |
|  |  | CC | 1,147 |  |
|  |  | CT | 163 |  |
|  |  | TT | 10 |  |

P<0.05 show statistical data with significant difference.
